# Experimental Study of a Deep-Learning RGB-D Tracker for Virtual Remote Human Model Reconstruction

**DOI:** 10.1155/2021/5551753

**Published:** 2021-09-15

**Authors:** Shahram Payandeh, Jeffrey Wael

**Affiliations:** Networked Robotics and Sensing Laboratory, School of Engineering Science, Simon Fraser University, Burnaby, British Columbia, Canada V5A 1S6

## Abstract

Tracking movements of the body in a natural living environment of a person is a challenging undertaking. Such tracking information can be used as a part of detecting any onsets of anomalies in movement patterns or as a part of a remote monitoring environment. The tracking information can be mapped and visualized using a virtual avatar model of the tracked person. This paper presents an initial novel experimental study of using a commercially available deep-learning body tracking system based on an RGB-D sensor for virtual human model reconstruction. We carried out our study in an indoor environment under natural conditions. To study the performance of the tracker, we experimentally study the output of the tracker which is in the form of a skeleton (stick-figure) data structure under several conditions in order to observe its robustness and identify its drawbacks. In addition, we show and study how the generic model can be mapped for virtual human model reconstruction. It was found that the deep-learning tracking approach using an RGB-D sensor is susceptible to various environmental factors which result in the absence and presence of noise in estimating the resulting locations of skeleton joints. This as a result introduces challenges for further virtual model reconstruction. We present an initial approach for compensating for such noise resulting in a better temporal variation of the joint coordinates in the captured skeleton data. We explored how the extracted joint position information of the skeleton data can be used as a part of the virtual human model reconstruction.

## 1. Introduction

Recent advancements in ambient sensing that combines visual and depth sensing modalities (RGB-D) have enabled investigation into their various potential applications for remote people tracking and activity reconstruction. Such systems can be integrated within the telemedicine framework for rehabilitation and mobility monitoring of the aging population. Historically, tracking of body limbs has been accomplished in a laboratory setup equipped with an IR sensor network where the subject can wear a collection of reflective markers attached. Through triangulation of a sensed location of markers, it is then possible to obtain the spatial locations of the markers with respect to a common coordinate frame. Such information is then correlated in order to reconstruct a stick-figure model of the connected body limbs (i.e., skeleton model) [1–5]. There are other types of wearable sensors which have recently been gaining popularity for estimating the main kinematic parameters of the movements of the whole body and its various limbs. These basic sensing modalities can consist of inertia measuring units [6], collection of RFIDs [7], or position sensing integrated within the exoskeleton devices [8].

**Figure 1:**
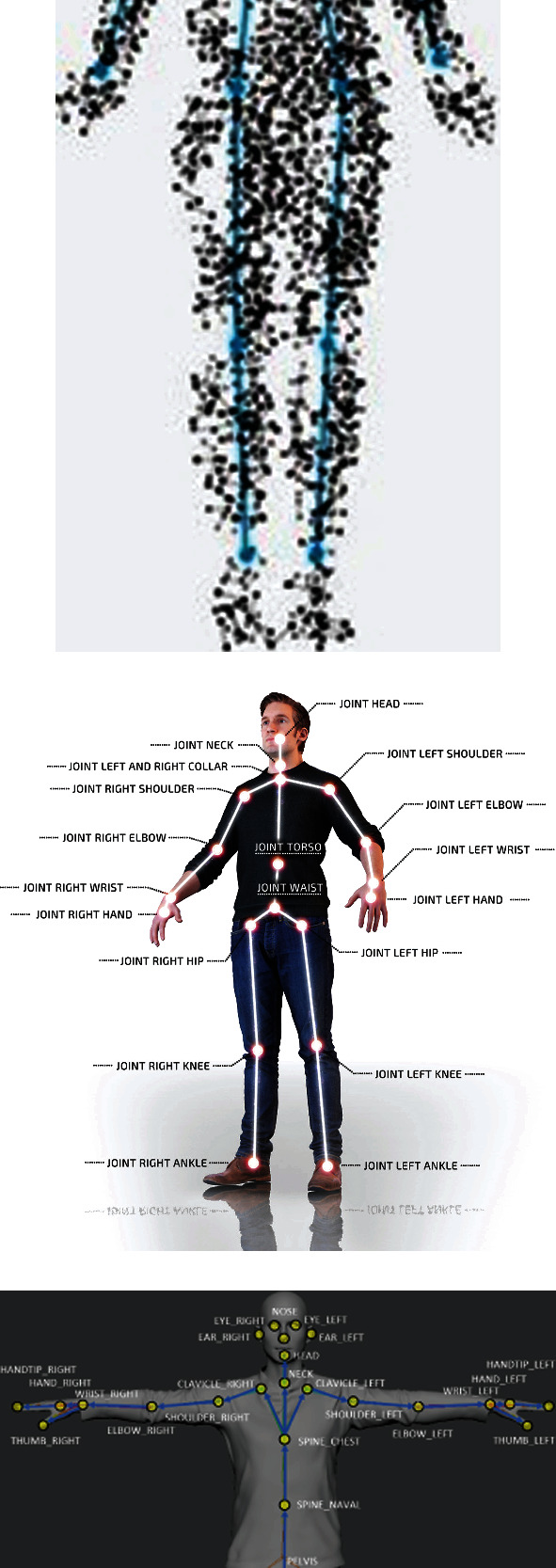
Tracking results of three body tracking algorithms: (a) Cubemos [9], (b) Nuitrack [10], and (c) Azure Kinect [11].

Recently, various commercially available deep-learning approaches for 2D body tracking have been proposed for RGB visual sensing alone. The results of these algorithms are then combined with depth sensing in order to further supply spatial information about the tracked subjects.  Figure 1 depicts three different ideal tracking results from three different commercially available algorithms. For example, Nuitrack [9] offers a deep-learning body tracker based on a 19-joint model representation. The system also offers some guidelines in creating the animated avatar model in the Unity environment. Another available deep-learning skeleton tracking algorithm is offered through Cubemos [10]. Like the Nuitrack algorithm, the main skeleton tracking scheme is accomplished through the utilization of RGB images of the subject. Depth data is used to further enhance the extracted coordinates of the joint locations of the stick-figure model which consists of an 18-joint skeleton model. Recently, Azure Kinect [11] has released another deep-learning SDK which proposes to track a 32-skeleton joint model under ideal monitoring conditions. In addition to the above, other similar research and developments are being proposed in only using multiple RGB signals [12] and methods based on using only depth data [13–14].

In this paper, we experimentally study the performance of one of the available and representative deep-learning body tracking algorithms, namely, Cubemos [10]. This algorithm is released through Intel RealSense RGB-D sensors [15]. We focus on the implementation of the sensor for tracking a person in an indoor environment. We study a typical performance of such a tracking scheme and challenges in associating the extracted skeleton coordinates for the reconstruction of an avatar model created in the Unity environment. Such virtual model reconstruction using an estimated sensed joint model can have many practical applications in remote monitoring and telehealth of older adults. These can be in tracking various gait of older adults during rehabilitation phases or in their activity recognition as related to dementia or development of serious games for their indoor exercises.

## 2. Application of Cubemos Body Tracker

**Figure 2:**
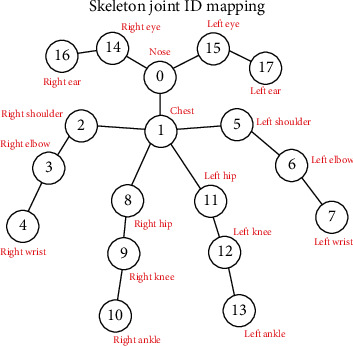
An 18-joint Cubemos [9] stick-figure model representing the skeleton model of the tracked subject.

In this section, we present a novel investigation into data obtained through the implementation of the Cubemos [10] body tracker algorithm. In this implementation and under ideal conditions, the information from the sensed data is mapped to a model depicted in Figure 2. The overall objectives are to record the sensed information obtained through visual and depth sensing in a natural indoor setting under natural illumination conditions, compute the stick-figure (skeleton) tracking data in real time, and reconstruct a virtual human model using the captured data.

Here, we first present some typical experimental results obtained by tracking the movements of a subject. It was found that in most cases, the tracking information can result in inconsistent variations of data or missing information. As a result, this can introduce challenges in any reconstruction attempt of the virtual human model based on the tracking data which can result in an unrealistic or distorted virtual model. Although there can exist many approaches where one can design and integrate various compensation algorithms, in the follow-up sections, we present some very basic approaches which can be used to obtain improved tracking and reconstruction results.

**Figure 3:**
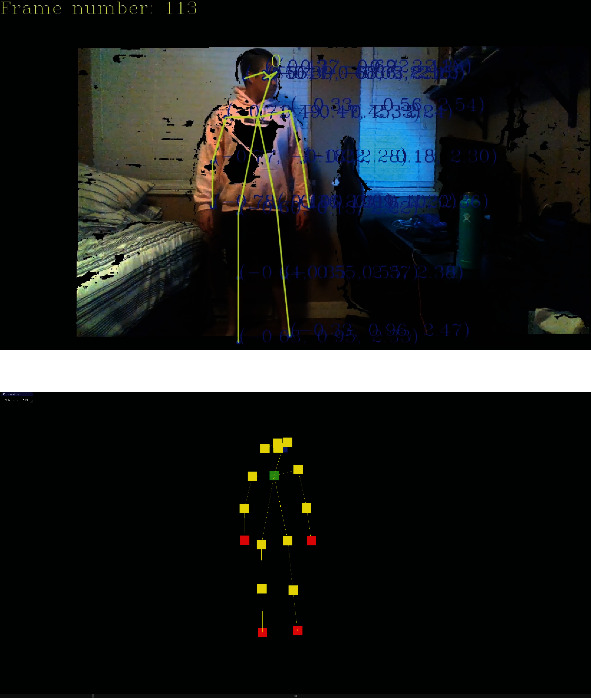
Initial processing of the captured sensor frame: (a) superimposed stick-figure model overlaid on top of the RGB image; (b) color-coded visualization of the skeleton model.

In an ideal tracking setup where the environment is properly lit and a person's whole body is visible, stationary (quasistatic), and facing the sensor, the skeleton tracking algorithm can perform well in constructing an estimated joint graph model for stationary locations of joints. For example, Figure 3 shows an overlaid joint graph model of a person in a room facing the sensor and looking to the side. As it can be seen in the captured frame of Figure 3, the tracker is easily able to locate most of the skeleton data (Figure 3(a)) except the joint associated with the left side of the head (joints 15 and 16 in Figure 2). By referring to Figure 2, these joints can further be clearly labelled using color-coded visualization where, for example, the blue joint is the nose (head), the green joint is the chest, and the red joints are the wrists and ankles (Figure 3(b)). The remaining yellow joints are the right ear and eye, shoulders, elbows, hips, and knees for a total of 18 mappable joints.

**Figure 4:**
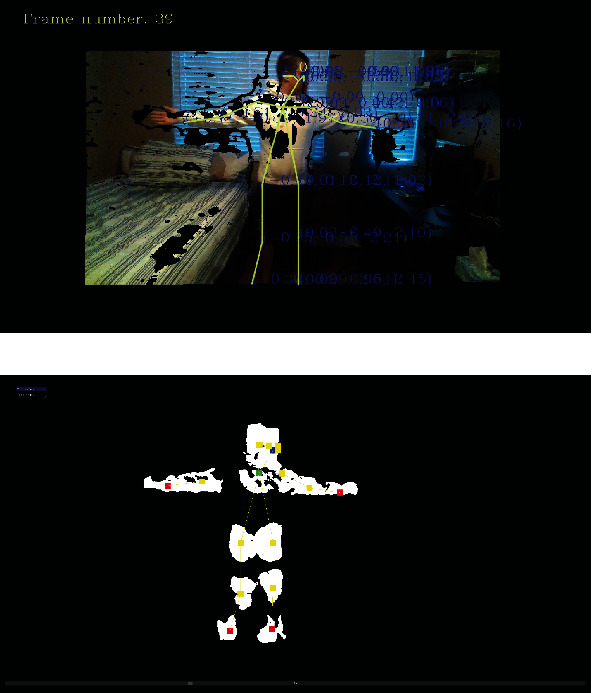
Tracked stick-figure information and point cloud segmentation: (a) overlaid tracked skeleton model on live visual image and (b) segmented point clouds associated with the corresponding definition of the skeleton joints and limbs. Information from such segmented point clouds is further utilized to develop a novel refinement of the tracked information associated with the locations of the skeleton joints.

Figure 4 shows a similar tracking frame to that of Figure 3 where all of the detected and tracked joints are shown with the corresponding additional segmented point clouds associated with the vicinity of the tracked skeleton joints and limbs, e.g., head, body torso, and the limbs. This segmented point cloud information was created and utilized as a part of the proposed novel graph-based estimation algorithm of this paper. This positional information associated with these segmented point clouds is used as a source of information to further estimate the missing joint data or to filter any noisy information during the movement tracking phase.

**Figure 5:**
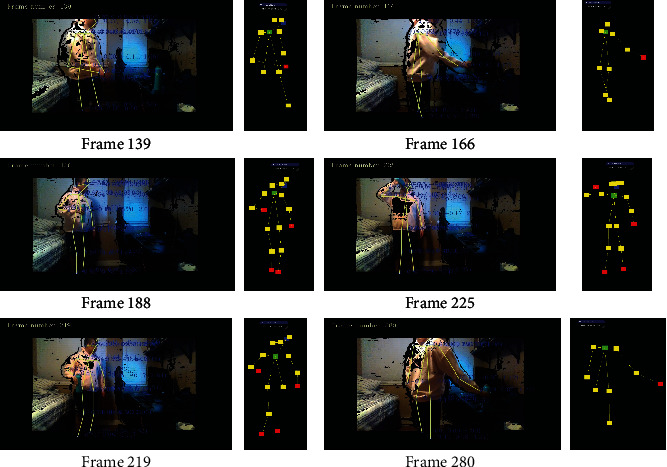
Various tracking frames showing an animated scenario of a subject reaching for a bottle, drinking from it, and placing it back on the desk. Each of the associated frames shows the results of overlaid 2D stick-figure tracking model and color-coded visualization of the captured 2D skeleton representation.

Next, we analyze the captured data for an animated scenario where the subject is performing a task. Under a natural illumination condition of a living space of a person, Figure 5 shows a case study where we have asked the subject to reach for a bottle of water located on a desk, drink from it, and then put it back on the desk. Figure 5 presents various instances of this task through various captured frames. The figure shows both the generated skeleton model on the actual RGB image and also a skeleton visualization of the captured images using color-based joint assignment.

**Figure 6:**
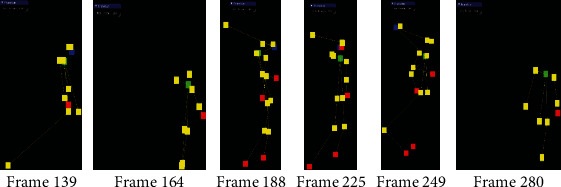
Visualization of the *z*-position error of the joints associated with the stick-figure model in the *y*‐*z* plane of the sensor coordinates of Figure 5.

As it is shown in Figure 5, the tracking information can be used to reconstruct the approximate representation of the skeleton model shown in the second and fourth columns. For example, the figure shows the right wrist (red joint) extended out, pulled back, brought near the nose joint, brought back down, and then extended back out. It was found that for a given sampled frame, the extracted *x*, *y*-coordinates of each skeleton joint offer smooth variations within the defined tracking frame. However, depth values of each joint (i.e., their *z*-coordinates or distance of each extracted joint with respect to the sensor) tend to have a high variance within their nominal values.  Figure 6 presents a visualization of such *z*-coordinate variations when viewed in the *y*‐*z* plane (where *y*-axis is in the downward direction, and *z*-axis is pointing outward from the sensor) of the captured frames associated within each of the subfigures shown in Figure 5. It can be observed that, for example, in frame 164 and for when the person is picking up the bottle, and in frame 280, for when the person is placing the bottle back on the table, the sensor has captured reasonable values for *z*-coordinate measurements for these joints. However, in the remaining frames, there are joints that are noticeably further away from the other joints in the skeleton, and clearly, their depth values are not accurate with respect to the anticipated locations on the RGB image.

**Figure 7:**
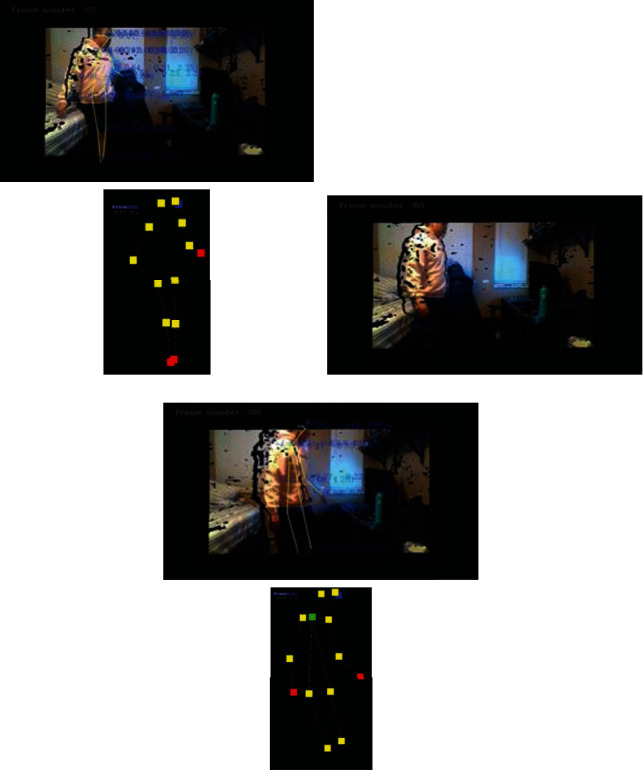
Examples of missing joint information when the view of the subject is self-occluded or different segments of the body are subjected to faster relative velocities.

In the cases when the person turns about its own axis and in the associated frames, the direct view of the joints which are being tracked is blocked and the tracking algorithm results in missing some or all the skeleton joints for in-between frames.  Figure 7 shows a close sequence of frames where a person is turning to sit on a chair. In the first set of frames, it can be observed that the chest and right wrist joints are missing even though they are clearly visible to the sensor in the live stream view. This is due to the properties of the deep-learning skeleton tracking algorithm which automatically discards any joints which are computed to have (0,0,0) coordinates. In the second set, there are no joints captured even though some are clearly visible in the live view, and in the third set, it is finally able to capture all the joints in view of the sensor.

## 3. Towards Enhanced Skeleton Tracking Method

In recent years, the development of deep-learning-based body tracking algorithms has offered a substantial leap forward in deploying a practical ambient sensing body tracker. However, these algorithms still required further enhancements to fully capture their potential practical applications in real living spaces. Depending on the algorithm, type of RGB-D sensor, and the conditions of the monitoring scene, additional compensation algorithms need to be designed and developed to estimate any missing tracking information. This is important when it is also required to reconstruct a virtual model of the subject based on the estimation of the joint locations of the skeleton model. Such reconstruction of the virtual model is an important component for telehealth and remote monitoring of the person which can protect the identity of the person and its surrounding environment and also allows visualization of the person while interacting with the environment. In this section and based on the initial observations of the tracking data presented previously, we explore a basic approach to compensate for any possible fast variations in the estimated joint coordinates. Although the development of general methods for such compensation is beyond the scope of the paper, the basis of our approach can be extended for any possible advanced estimation and filtering methods which also have been proposed in various other single tracking applications (e.g., [16, 17]).

Like any standard estimation algorithms, a reference frame is needed at the initialization frame for determining the initial joint coordinates of a pose. The information from the initial reference frame and the corresponding segmented point clouds associated with each joint and associated limb is used to assist in estimating the missing joint coordinates in the consecutive tracking frames. In our study, a default standing pose is used where the subject holds the arms by the side and legs together and is positioned in the direction that the sensor is facing, ensuring that all joints are visible by the sensor. Another important parameter which is used is the number of frames. In this initial approach and for defining the joint coordinates in the initial frame, standard deviations of all the depth coordinates of the body joints are calculated and the frame with the lowest standard deviation is defined as the initial frame for the follow-up estimation algorithm. Given the initial reference frame, the algorithm then computes the limb distances and the distances between the joints and the parent joints. Using the data structure of Figure 2, the parent joint is the joint that is connected to the root joint, which is the nose joint, either directly or via a chain of connections through other joints. This means that every joint will have a limb distance except for the nose/root joint.

Joints in a frame are processed in the order of the skeleton joint IDs, so the nose will be the first joint to be processed in the current frame. It checks if the joint has an acceptable displaced distance from the nose joint defined in the previous frame (within the defined lower and upper bounds). If the distance is within this bound, the current frame coordinates of the joint are kept, and the algorithm would move on to the following skeleton joint ID. If the displaced distance is not within the bound (i.e., within the limb distance), then coordinates of the current joint are to be modified through the estimation algorithm.

In our proposed method, the algorithm first searches the segmented point cloud coordinates of the frame associated with the missing joint (an example of such segmentation is shown in Figure 4(b)). If the nose/root joint is missing, then it utilizes the associated segmented point cloud point closest to the coordinates of the nose joint in the previous frame used. If one of the remaining joints is missing, it selects the closest point cloud point to the joint coordinates of the previous frame that is also limb distance away from its parent joints. If the distance between this new point cloud point and the current frame joint is within the bound, the new coordinate of the joint is set to mean values between the newfound point cloud point and the current frame joint. If this distance is not within the current joint bounds, the new coordinate of the joint is set at the newfound point cloud point coordinates. The algorithm will also adjust the coordinates to the mean value point between the newfound point cloud point and the current frame joint if the distance to the joint in the previous frame is decreased because of making this adjustment.

### 3.1. Sample Results

**Figure 8:**
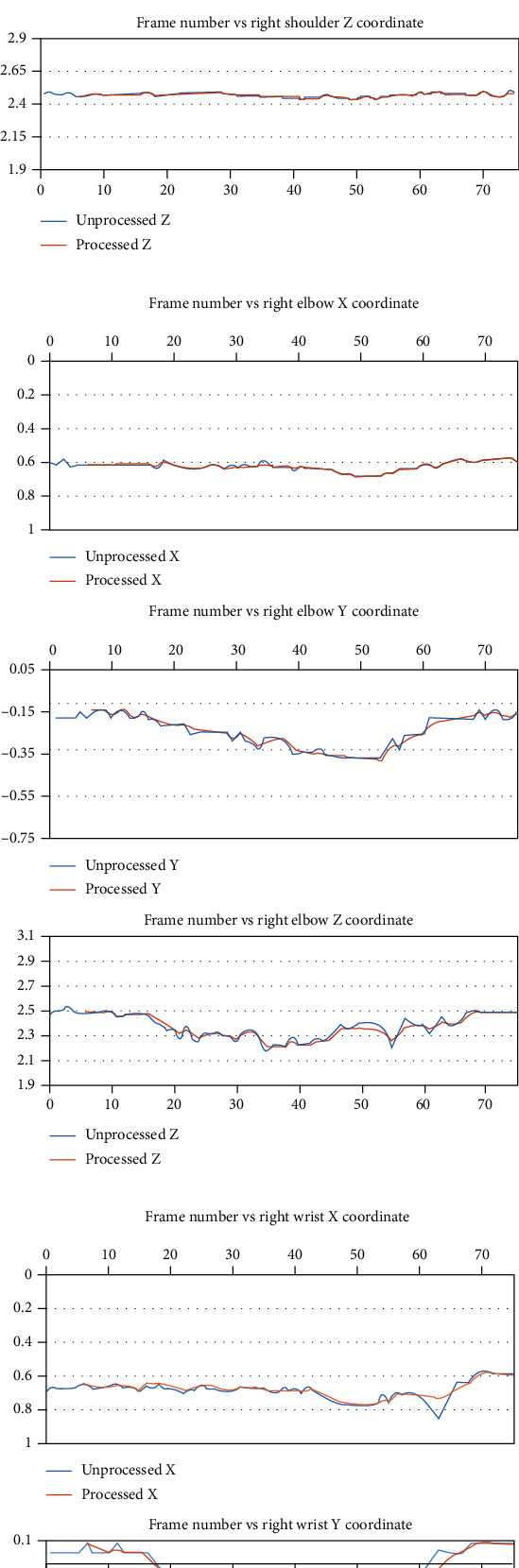
Sample plots of the filtering/estimation results obtained from the deep-learning body tracker for the (a) right shoulder; (b) right elbow, and (c) right wrist coordinates.

**Figure 9:**
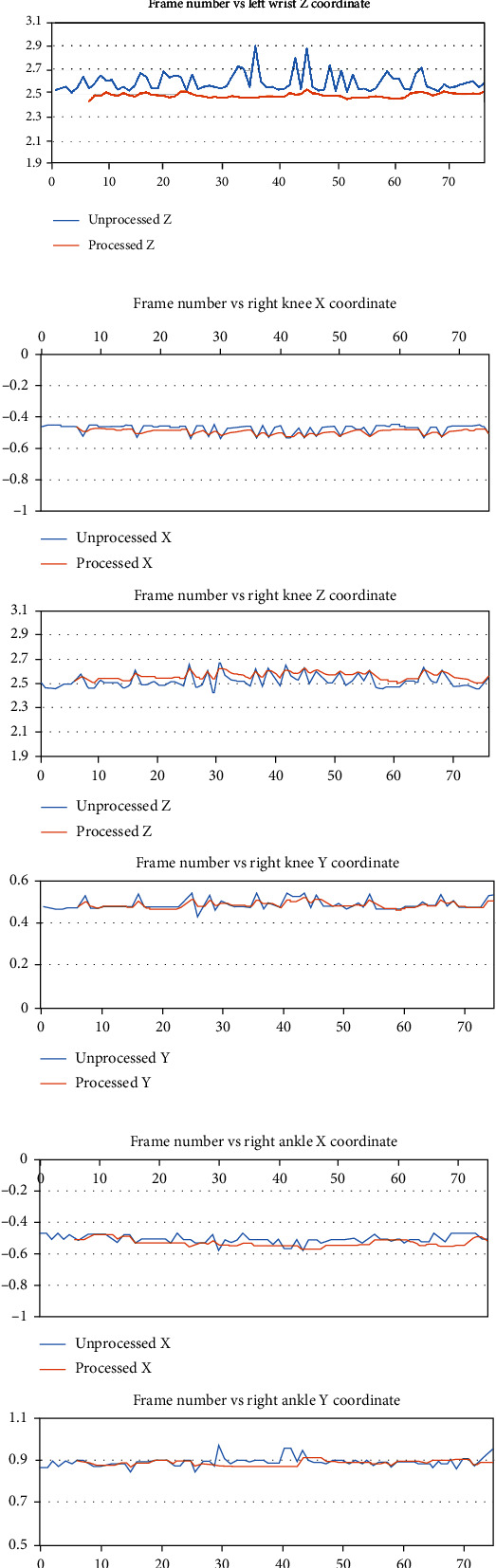
Sample plots of the estimation/filtering results obtained from the deep-learning body tracker for the (a) left wrist, (b) right knee, and (c) right ankle coordinates.

**Figure 10:**
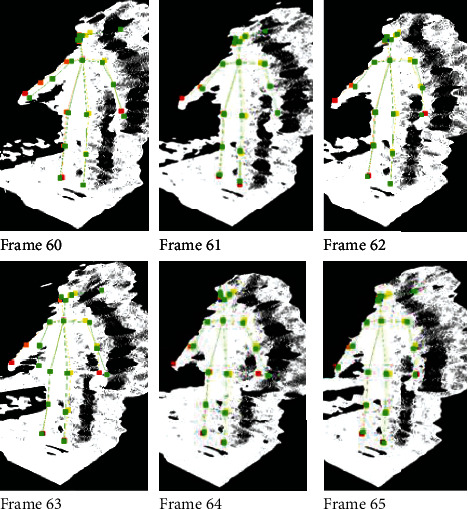
Visualization of the proposed point cloud compensation algorithm based on the deep-learning body tracker model for a sample case where the right wrist joint is missing in frames 61, 62, and 64 and the case where the right wrist joints have an inaccurate depth value. Unprocessed skeleton data are in green, and the estimated skeleton data are shown in multiple colors.

For our test procedure, while facing the sensor, the person is only moving up from the side and then back down. The algorithm is easily able to correct any of the skeleton joints with large variations in the depth values. Figures 8 and 9 show sample plots for both unprocessed and processed joint tracking data. Frames 60 to 65 in Figure 10 show the visualized examples of the algorithm handling a case where the right wrist joint is missing in frames 61, 62, and 64 and the case where the right wrist joints have an inaccurate depth value.

The visualizer in Figure 10 shows the unprocessed skeleton data in green and the processed skeleton data in multiple colors. Focusing on the right wrist of the skeleton, the red joint, we can see that it successfully followed the actual movement of the right wrist moving downward, depicted by the point cloud points of the right arm.

## 4. Enhanced Skeleton Data for Virtual Human Model Reconstruction

Having the 3D coordinates of each extracted joint of the skeleton model, the challenge is on how to map these coordinates to the virtual human model to reconstruct the joint movements for a given posture. The data structure associated with the construction of the virtual model in general takes advantage of the relative coordinate frame definitions which are commonly used in the core OpenGL (or similar API) implementation.

**Figure 11:**
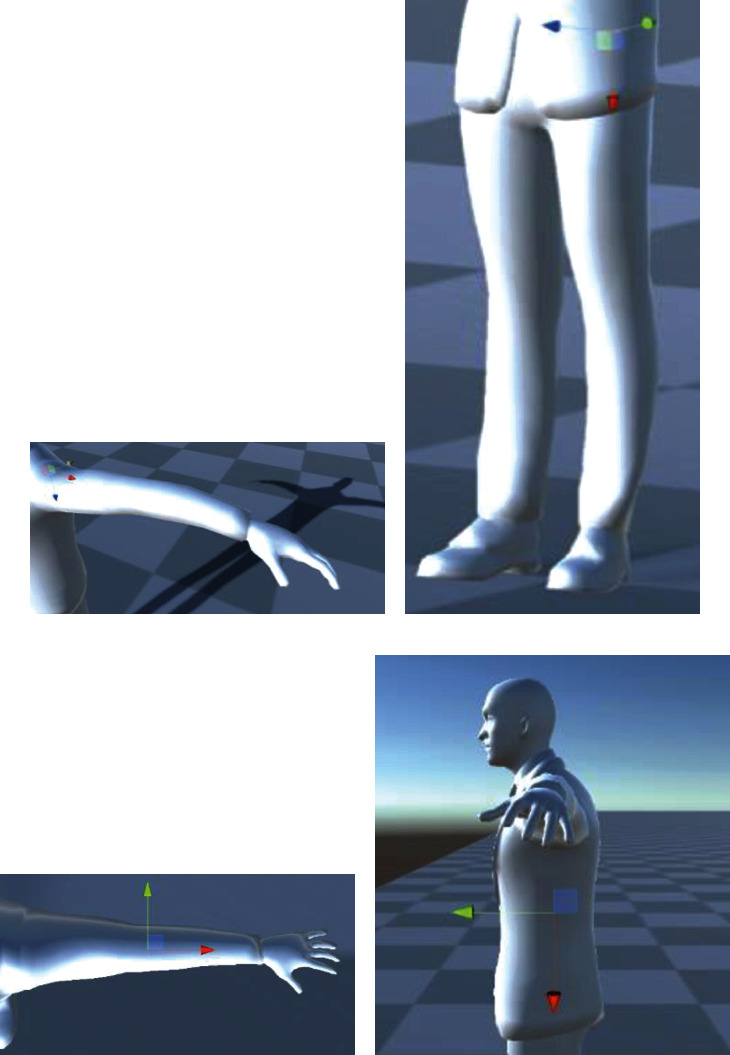
Examples of coordinate frame assignments on a virtual human model: (a) shoulder joint, (b) hip joint, (c) elbow joint, and (d) vertebral body coordinate. The *x*-axis is shown in red, the *y*-axis is shown in green, and the *z*-axis is shown in blue.

In Figure 11, the *x*-axis is shown in red, the *y*-axis is shown in yellow, and the *z*-axis is shown in blue. Each joint position of the virtual model is defined by a right-handed Cartesian frame representation. Using the extracted and refined skeleton model, the relative position of the origins of these frames can be defined with respect to the position of their parent nodes.

**Figure 12:**
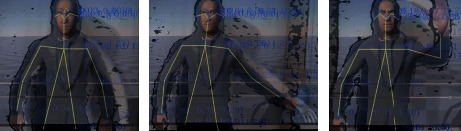
Examples of skeleton tracker model superimposed on the live image of the person which is also overlaid on the virtual human model.

Figure 12 shows examples of the skeleton tracker model superimposed on the live image of the person which is also overlaid on the virtual human model. The example frames are associated with the person standing in front of the sensor and raising the left arm to the side and over the head.

The kinematic construction of the virtual human is based on a simplified representation of various links and joints which can then be used by the graphical model. In our model, the movements of the head (e.g., flexion, extension, lateral bending, and rotation), hip (flexion, extension, and abduction), and shoulder joints (e.g., abduction and horizontal and vertical flexion) are modeled as spherical joints. Elbow (e.g., extension, flexion, and supination/pronation) and knee (flexion and extensions) joints are modeled as turning (revolute) joints. For the humanoid model, a spherical joint is also used to represent the relative movements of the upper body with respect to the hip.

The main challenges in mapping the tracked skeleton coordinates obtained from deep-learning-based approaches to the virtual human model reconstruction are the ambiguities they present in resolving the kinematic joint angles of the virtual model. For example, referring to Figure 2, the right and left arms correspond to the coordinates of the skeleton joints of (2, 3, and 4) and (5, 6, and 7), respectively. From this information, one needs to associate their relative positions to the corresponding kinematic joint angles in the virtual model reconstruction. Having been able to resolve some of these mappings for various frames (i.e., key frames), we can use various trajectory interpolation algorithms (available through Unity) to animate for in-between frames. To initialize the Unity model with respect to the skeleton tracking algorithm, the initial captured data is used for computing the relative position of each joint coordinate frame. In the following, we present some examples of the proposed kinematic model extraction for resolving ambiguities for virtual human model reconstruction. In the following, we present some practical implementation details of how these angles are computed.

### 4.1. Shoulder Joints

**Figure 13:**
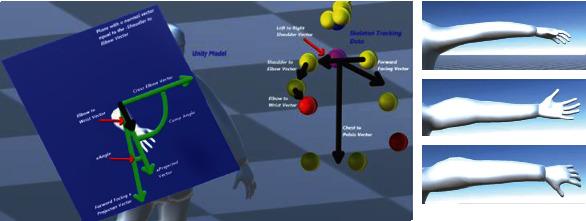
Geometrical description of the shoulder joint for computation of rotation angle about the *x*-axis (circumduction) using skeleton tracking data.

**Figure 14:**
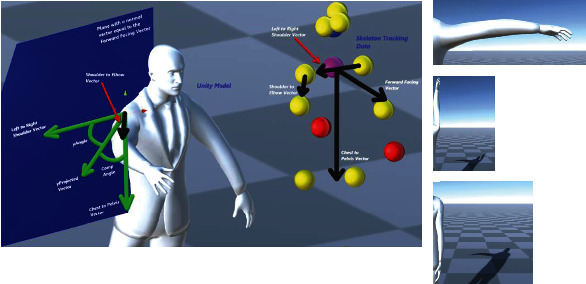
Geometrical description of the shoulder joint for computation of rotation angle about the *y*-axis using the skeleton tracking data.

**Figure 15:**
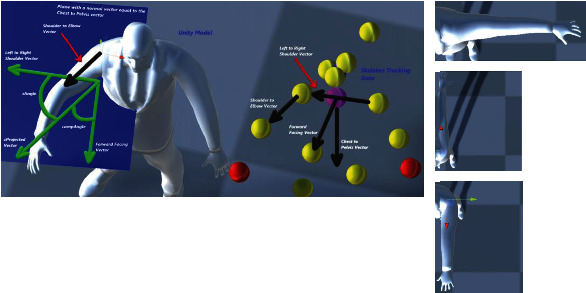
Geometrical description of the shoulder joint for computation of rotation angle about the *z*-axis (flexion and extension) using the skeleton tracking data.

For resolving a shoulder joint for a given relative position of the connected elbow joint obtained from the skeleton tracking data, we utilized the inverse Euler angle solution approach [18]. These angles are interpreted with respect to the local coordinate frame which is defined at the parent location of the elbow joint (see Figures 13–15). These extracted angles are then directly mapped to the script of the kinematic model of the virtual human in Unity.

One of the Euler angle parameters is the rotation around the local *x*-axis which rotates the arm by twisting it either behind or towards the front of the body (circumduction).  Figure 13 is a visualization of the vectors used to calculate this angle. The black arrows show the relative position vectors calculated from the global coordinates of the skeleton tracking data. We compute the cross product between two vectors defined by joints like “Left to Right Shoulder” vector and the “Chest to Pelvis” vector (the pelvis joint being the created joint placed in the middle of the 2 hip joints) (Figure 2) to find the “Forward Facing” vector. Then, we project the “Forward Facing” vector onto the plane with a normal vector equal to the negative “Shoulder to Elbow” in order to compute the “Forward Facing x Projection” vector. The “Shoulder to Elbow” vector is then projected on the same plane in order to obtain the “xProjected” vector. In Figure 13, all the vectors shaded in green are located on this plane, which is also shaded in blue. One more reference vector is found by taking the cross product of the “Forward Facing x Projected” vector and the negative “Shoulder to Elbow” vector to obtain the “Cross Elbow.” The rotation about the local *x*-axis is then set to be equal to the angle between the “xProjected” vector and the “Forward Facing x Projected” vector. This will result in the computed angle to be always positive. In order to adjust this value which can also include the negative sense of rotation, we check if the “xProjected” vector is in between the “Cross Elbow” vector and the “Forward Facing x Projected” vector or if the “xProjected” vector is in between the “Cross Elbow” vector and the negative “Forward Facing x Projected” vector; otherwise, the angle is kept positive.

Similarly, Figure 14 shows the geometry which is used for computing the rotation about the *y*-axis (abduction and adduction). The black arrows show the relative vectors calculated from the global coordinates of the skeleton tracking data. We take the cross product of the “Left to Right Shoulder” vector and the “Chest to Pelvis” (the pelvis joint being the created joint placed in between the 2 hip joints) to find the “Forward Facing” vector. The “Shoulder to Elbow” vector is then projected on the plane with a normal vector equal to the “Forward Facing” vector to get the “yProjected” vector. In Figure 14, all green vectors are on this plane, which is shaded in blue. The yAngle is then set to equal the angle between the “yProjected” vector and the “Left to Right Shoulder” vector; however, this angle will always be a positive value, but we want to adjust the angle such that the angle is made negative if the “yProjected” vector is in between the “Chest to Pelvis” vector and the “Left to Right Shoulder” vector or if the “yProjected” vector is in between the “Chest to Pelvis” vector and the negative “Left to Right Shoulder” vector; otherwise, the angle is kept positive.

Figure 15 shows the geometrical model which is used for computing the rotation about the *z*-axis of the shoulder joint (flexion and extension). The angle rotates the joint in a way that moves the arm in front of the body or moves behind the body. The black arrows show the relative vectors calculated from the global coordinates of the skeleton tracking data. We take the cross product of the “Left to Right Shoulder” vector and the “Chest to Pelvis” (the pelvis joint being the created joint placed in the middle of the 2 hip joints) to find the “Forward Facing” vector. The “Shoulder to Elbow” vector is then projected on the plane with a normal vector equal to the “Chest to Pelvis” vector to get the “zProjected” vector. In the figure, all the green vectors are on this plane, which is shaded in blue. The zAngle is then set to equal the angle between the “zProjected” vector and the “Left to Right Shoulder” vector; however, this angle will always have a positive value which can be adjusted for its clockwise and counterclockwise sense of rotation.

### 4.2. Hip Joint

**Figure 16:**
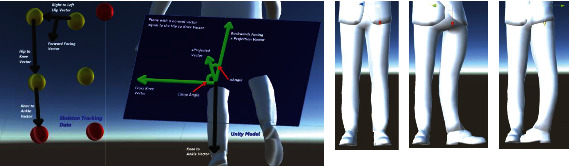
Geometrical description of the shoulder joint for computation of rotation angle of the hip joint about the local *x*-axis (external and internal rotation) using the skeleton tracking data.

A similar ambiguity resolution was followed for the reconstruction of the kinematic angles of the hip joints with respect to the local *x*-, *y*-, and *z*-coordinates. In the following, we present some implementation details of how these angles were computed. In Figure 16, black arrows show the relative positional vectors computed from the global coordinates of the skeleton tracking data. The vector product between the two vectors “Right to Left Hip” and “Global Up” results in finding the “Forward Facing” directional vector. We project the negative of the “Forward Facing” vector onto the plane with a normal vector equal to the negative “Hip to Knee” vector to compute the “Backwards Facing x Projection” vector. The “Knee to Ankle” vector is then projected on the same plane to get the “xProjected” vector. In the figure, all the green vectors are on this plane, which is shaded in blue. An additional reference vector is found by computing the vector product of the negative “Hip to Knee” vector and the “Backwards Facing x Projection” vector to obtain the “Cross Knee” vector. The xAngle is then set to equal the angle between the “xProjected” vector and the “Backwards Facing x Projected” vector; however, this angle will always be a positive value, but we want to adjust the angle such that the angle is made negative if the “xProjected” vector is in between the “Cross Knee” vector and the “Backwards Facing x Projection” vector or if the “xProjected” vector is in between the “Cross Knee” vector and the negative “Backwards Facing x Projection” vector; otherwise, the angle is kept positive.

**Figure 17:**
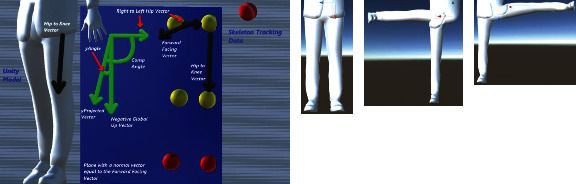
Geometrical description of the shoulder joint for computation of rotation angle of the hip joint about the local *y*-axis (abduction and adduction) using the skeleton tracking data.

**Figure 18:**
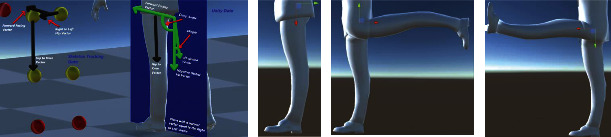
Geometrical description of the shoulder joint for computation of rotation angle of the hip joint about the local *z*-axis (flexion and extension) using the skeleton tracking data.

Similarly, Figures 17 and 18 show examples of geometrical models for computing the rotation of the hip joint about the *y*- and *z*-axes.

### 4.3. Elbow Joint

**Figure 19:**
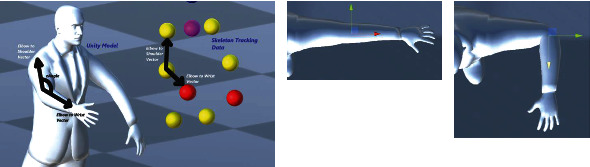
Local skeleton data which are used in computing the local Euler angle rotation about the *z*-axis of the elbow coordinate frame (flexion and extension).

The virtual kinematic model of the elbow joint is represented as a single revolute joint (i.e., rotation about local *z*-axis). Its magnitude is found by determining the angle between the vectors obtained by “Elbow to Shoulder” and “Elbow to Wrist” vectors (flexion and extension).  Figure 19 shows the geometrical model which is used for the computation of the elbow angle as a function of the skeleton data.

### 4.4. Knee Joint

**Figure 20:**
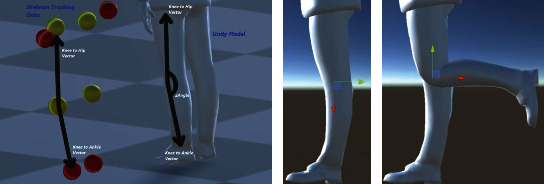
Local skeleton data which are used in computing the Euler angle rotation about the *z*-axis of the knee coordinate frame (flexion and extension).

The computation for the knee joint based on the skeleton tracking data for kinematic model reconstruction is like the elbow joint, and it is based on rotation about a revolute joint and the local *z*-axis. Its rotation magnitude is found by computing the angle between the vectors that correspond to the “Knee to Hip” and “Knee to Ankle” vectors.  Figure 20 is a visualization of the calculation based on the geometrical data of the skeleton model (flexion and extension).

### 4.5. Vertebral Column Joint

**Figure 21:**
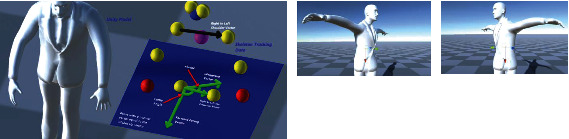
Geometrical description based on the skeleton data for computing the vertebral column joint rotation about the local *x*-axis.

The rotation around the local *x*-axis causes the upper body to rotate clockwise or counterclockwise. The angle is found by first finding the projection of the “Right to Left Shoulder” vector onto the plane with a normal vector equal to the “Global Up” vector. This results in the “xProjected” and the “Right to Left Hip” vectors projected onto the same plane to compute the “Right to Left Hip Projection” vector. In Figure 21, all the green vectors are on this plane, which is shaded blue. Another reference vector is defined by taking the cross product of the “Right to Left Hip” vector and the “Global Up” vector to get the “Forward Facing” vector. The xAngle is then set to equal the angle between the “xProjected” vector and the “Right to Left Hip Projection” vector.

**Figure 22:**
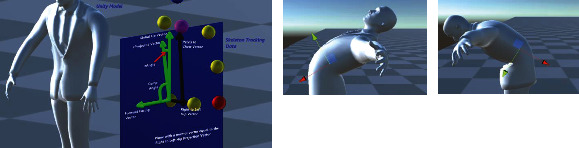
Geometrical description based on the skeleton data for computing the vertebral column joint rotation about the local *z*-axis (extension and flexion).

The rotation around the local *z*-axis is the extension and flexion which make the body bend forward and backwards. The angle is computed by first finding the projection of the “Right to Left Hip” vector onto the plane with a normal vector along the “Global Up” vector to get the “Right to Left Hip Projection” vector. Then, the “Pelvis to Chest” vector is projected onto the plane with a normal vector aligned with the “Right to Left Hip Projection” vector to get the “zProjected” vector. In Figure 22, all the green vectors are on the plane with a normal vector aligned to the “Right to Left Hip Projection” vector, shown as shaded blue. One more reference vector is found by taking the cross product of the “Right to Left Hip” vector and the “Global Up” vector to get the “Forward Facing” vector. The rotation angle is then set to equal the angle between the “zProjected” vector and the “Global Up” vector.

## 5. Conclusions

Tracking movements of the body using ambient sensing in a natural living environment can offer many advantages for various health monitoring and telemedicine systems. Such setup can allow the subject to freely move around without wearing any specialized sensors. Recent advancements and availability of commercial body tracking systems using RGB-D sensing have offered some promises in achieving the above objectives. The implementation of these body tracking systems is based on various deep-learning methodologies where they offer various body landmarks on the image of the tracked subject in a form of joint and link placements (i.e., skeleton model). Given this tracked information, it can then be possible to reconstruct the virtual human model using some off-the-shelf design tools. Playbacks of the virtual human model in the VR of the living space can protect the privacy of the subject while allowing visualization of movements and activities of the subject.

In this paper, we experimentally study the performance of one of these deep-learning approaches for body tracking. We further explored the challenges of how this skeleton joint information can be used to resolve and reconstruct the graphical model of the subject using standard kinematic model description. This paper presented some preliminarily experimental studies and analyses of integrating one of these commodity-based sensors (namely, Intel RealSense D435) and the associated deep-learning body tracker (namely, Cubemos) in a natural living environment. The paper further presents results of ambiguity resolution of associating the position of the skeleton joint data for the kinematic joint angle reconstruction of the virtual human model.

Through our study, we were able to show that the current implementation in extracting the movements of the limbs of the body still requires an additional layer of signal processing to alleviate the existence of noise (for example, associated with the depth coordinate). We have shown some basic approaches for obtaining a smoother transition of the tracking data for in-between frames. However, one of the challenges of future work is the design and development of a robust intelligent layer which further incorporates the existing skeleton tracking data that can be used in enhancing or compensating for any missing information. Such missing tracking data can be caused by various cases of occlusions and self-occlusions of the body or the presence of another person or pieces of furniture in the scene [19].

Reconstruction of the virtual human body from the sparse tracking data is another main challenge. Kinematic modeling of the human body can be accomplished through the definition of various coordinate frames. The relative joint movements of the limbs can be defined in various local frames. However, associating the 3D coordinates of the tracking skeleton data to that of the kinematic model can present several challenges. This paper presents some initial approaches for associating the data obtained from the tracking information to that of the virtual human model reconstruction.

## Data Availability

Associated data with the segmented and reconstructed scenes is available from the authors.
